# Beyond static biomarkers: systems biology and AI for decoding cancer dynamics

**DOI:** 10.3389/fsysb.2026.1855016

**Published:** 2026-07-14

**Authors:** Subhajit Dutta, Anjana Goli

**Affiliations:** 1 Department of Biochemistry and Molecular Cell Biology (IBMZ), Center for Experimental Medicine, University Medical Center Hamburg-Eppendorf, Hamburg, Germany; 2 Department of Molecular and Cell Biology, University of California, Berkeley, CA, United States

**Keywords:** adaptive therapy, artificial intelligence, cancer dynamics, cell states, dynamic biomarkers, liquid biopsy, machine learning, mathematical oncology

## Abstract

Precision oncology has been built largely on static biomarkers, including mutational profiles, receptor status, histopathologic classes, and single-time-point molecular signatures. These readouts have transformed diagnosis and treatment selection, but they remain mismatched to a disease whose most consequential behaviors—progression, metastasis, treatment adaptation, dormancy, and relapse—are dynamic, noisy, and multiscale. In this article, we argue that cancer is better understood as a stochastic, coupled biological system than as a fixed molecular identity. We bring together adjacent literatures spanning cancer cell states, spatial and ecological organization, metabolism and dormancy, mechanobiology, longitudinal biomarkers, quantitative oncology, and AI-enabled representation learning, and develop a mathematically grounded framework for reasoning about cancer dynamics. Our central claim is modest but consequential: future biomarkers should not only classify current disease state, but also estimate transition risk, system instability, and trajectory direction. To support that claim, we distinguish latent biological state from clinical observation, clarify why partial observability makes dynamic inference difficult, and introduce a mathematically explicit but deliberately constrained formal scaffold based on stochastic state-space models, local linearization, and layer-specific dynamical motifs. We then develop six internal biological layers of cancer dynamics—molecular regulatory dynamics, cellular state plasticity, spatial niche organization, tumor ecosystem co-evolution, metabolic-epigenetic coupling with dormancy, and mechanobiological feedback—and treat longitudinal clinical monitoring as a linked observation layer rather than a mechanistic subsystem. Particular attention is given to noise; transcriptional noise, ecological variability, treatment-induced perturbation, and measurement noise all shape how cancer states are occupied, destabilized, and detected. Finally, we review dynamic biomarker evidence from ctDNA-guided adjuvant therapy, circulating tumor cells, serial imaging, and adaptive therapy, discuss how AI can support inference under partial observability without replacing mechanism, examine regulatory and health-equity constraints, and outline the research agenda needed to turn dynamic oncology from a compelling idea into a reproducible clinical discipline.

## 1 Introduction

The modern precision-oncology era was built on a simple and powerful assumption: if tumors could be molecularly characterized with sufficient granularity, treatment could be matched to disease biology. In practice, that assumption enabled genuine progress—genomic alterations, receptor status, immune markers, and pathology features now guide diagnosis, risk stratification, and therapeutic choice across many cancers. Yet the limitations of that framework are equally visible. Tumors with similar baseline molecular profiles often follow different clinical courses; initially responsive cancers adapt under therapy, disseminated cells can persist for years before reactivation, and radiographic or circulating biomarkers often lag behind the underlying biological transition that eventually becomes clinically obvious (Nowell, 1976; Hanahan, 2022; Passaro et al., 2024).

The common feature across these failures is not a lack of molecular detail. It is a mismatch between cross-sectional measurement and time-dependent disease. Cancer progression is driven by coupled processes that unfold across regulatory networks, cellular state transitions, spatial niches, ecological competition, metabolic adaptation, mechanical feedback, and clinical intervention. These processes do not simply coexist; they constrain one another. A systems-biology account of cancer, therefore, requires more than an expanded parts list. It requires a framework for reasoning about how biological state changes, how stochasticity reshapes that change, and how partial clinical observations can or cannot reveal it (Wiley et al., 2025; Tirosh and Suva, 2024; Merlo et al., 2006).

This review differs from the adjacent literature in two key respects: it extends beyond state description to cross-scale coupling and places dynamic biomarkers and clinical observation at the center of the argument rather than at the periphery (Tirosh and Suva, 2024; Wiley et al., 2025; Passaro et al., 2024). Learning-based methods are treated primarily as tools for inference under partial observability rather than as ends in themselves (Yates and Van Allen, 2025; Riaz et al., 2025).

Because the present synthesis sits close to several recent reviews, it is worth stating precisely what it adds to each. Tirosh and Suva (2024) provide an authoritative account of how single-cell data define and stabilize cancer cell states, but their focus is the characterization of states rather than the observation process through which states are clinically inferred or the coupling of the cellular layer to others; here, cell-state plasticity is treated as one layer within an explicitly coupled, partially observed system (Section 4.2; Equation 5). Passaro et al. (2024) survey longitudinal and dynamic biomarker modalities, particularly in the liquid-biopsy setting, and catalogue what can be measured serially; the contribution here is not an expanded catalogue of modalities but a formal distinction between the latent biological state and its clinical observation (Equations 1, 2), which is what allows a biomarker change to be interpreted as a true transition, a delayed observation, or an artifact. Holder et al. (2024) address the clinical utility of immune-checkpoint biomarkers and the gap between a biomarker that predicts and a biomarker that should change management; we adopt that validity-versus-utility distinction (Section 6.6) but embed it in a multiscale dynamical framing in which the layer driving a detected transition determines whether the chosen intervention is mechanistically matched to it. In short, where these sources respectively characterize states, enumerate dynamic modalities, and adjudicate clinical utility, this review’s distinct contribution is the integration of all three under a single observation-aware, cross-layer dynamical scaffold.

Why is this the right moment for a synthetic review of cancer dynamics? Because several once-separate literatures have matured enough to demand integration. Single-cell studies have stabilized the language of cancer cell states; spatial omics has made tissue geography measurable; ecological and evolutionary oncology has linked resistance to competition and selection; and ctDNA-guided trials have begun to test biomarker-triggered intervention (Tirosh and Suva, 2024; Liu et al., 2026; Gatenby and Brown, 2020; Tie et al., 2022; Tie et al., 2025a). Quantitative modeling now spans from mechanistic ODEs to probabilistic latent-state models and foundation models such as scGPT and Geneformer (Yates and Van Allen, 2025; Cui et al., 2024; Theodoris et al., 2023). Measurement maturity now exceeds conceptual integration.

**FIGURE 1:**
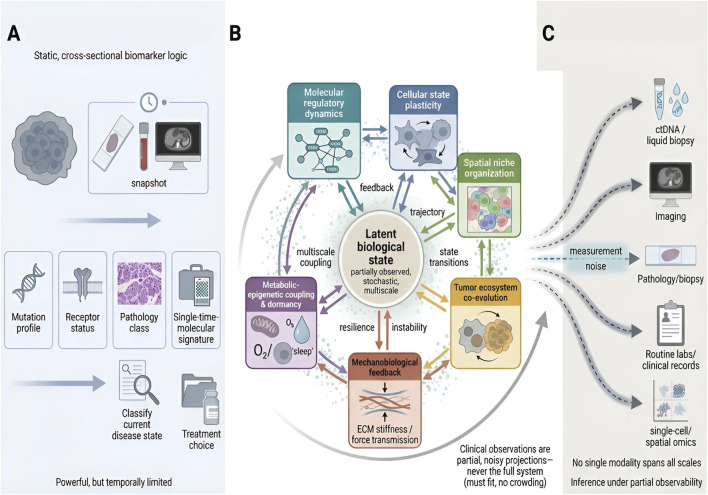
From static biomarkers to stochastic cancer dynamics. Conceptual overview of the review framework. Panel **(A)** summarizes conventional cross-sectional biomarker logic. Panel **(B)** depicts cancer as a stochastic multiscale dynamical system with six interacting internal biological layers. Panel **(C)** emphasizes that clinical assays observe only partial and noisy projections of that latent biological state.

The aim of this article is therefore twofold. The primary aim is biological: to provide a clear systems-biology synthesis of cancer as a multiscale dynamic disease. The secondary aim is formal: to offer a quantitative language that is specific enough to organize hypotheses, local stability reasoning, and biomarker design, but restrained enough to avoid pretending that a universal predictive model of cancer already exists. In that sense, this article is best read as a perspective rather than an exhaustive review. Although we draw on established literatures across single-cell biology, evolutionary oncology, spatial omics, and computational modeling, the six-layer architecture, the composite state vector (Equation 10), the evidence-tier ranking, and the block-Jacobian coupling analysis (Equation 11) are original analytical contributions of this article rather than summaries of prior frameworks. The mathematically explicit scaffold is intended to organize hypotheses and clarify the biology, not to define a universal predictive model. The overall architecture of the argument is summarized in Figure 1.

### 1.1 Scope and literature approach

This article is a narrative, hypothesis-organizing perspective. The literature was assembled through database searching and citation chaining across stochastic systems thinking, multiscale cancer dynamics, longitudinal biomarkers, and computational inference. Priority was given to mechanistic studies, landmark trials, and synthetic reviews; the goal was conceptual integration rather than exhaustive enumeration.

## 2 A systems-biology vocabulary for cancer dynamics

The phrase “cancer dynamics” is often used loosely. In this review, we use it in a stricter sense. Cancer dynamics refers to time-dependent change in tumor state across interacting biological scales, where that change is structured, noisy, coupled, and shaped by feedback. Three distinctions are especially important. A state is the current configuration of the system or a subsystem. A transition is a shift from one state to another. A trajectory is the broader path by which states evolve through time. Instability, as used in this review, refers specifically to loss of local stability in the dynamical-systems sense: a condition in which the dominant eigenvalue of the local Jacobian approaches zero, making the current operating point increasingly vulnerable to perturbation. Much of biomarker science still emphasizes state recognition, whereas the hardest clinical problems—treatment escape, relapse, metastatic awakening, and loss of response—depend on transition risk and trajectory direction (Passaro et al., 2024; Tirosh and Suva, 2024; Holder et al., 2024).

A second distinction is between observable and inferred dynamics. Much of dynamic oncology is an inverse problem: investigators observe a limited projection of the disease and attempt to infer the latent biological state that generated it. The observation process is a defining constraint on what dynamic claims can be justified (Nguyen Phuong et al., 2025; Tie et al., 2022).

A third distinction is between multiscale coexistence and multiscale coupling. The harder question is how change at one scale propagates to another: regulatory rewiring reshapes cellular-state accessibility, spatial structure channels ecological interaction, and mechanical forces alter drug delivery. The challenge is to specify the couplings, not simply list the scales (Wiley et al., 2025; Yuan, 2016; Jain et al., 2014).

### 2.1 Noise is not an accessory variable

Noise is central to this framework. In cancer, noise includes transcriptional bursting, chromatin-state fluctuation, uneven perfusion, variable immune contact, and stochastic state switching. Together, these fluctuations determine how frequently a population samples alternative states, how sharply a transition boundary is experienced, and how early a clinical assay can detect motion. Noise is both a driver of cancer dynamics and a readout of the same processes (Gupta et al., 2011; Mojtahedi et al., 2016; Huang et al., 2009).

Because the term “noise” is used throughout this review for several phenomena that are not interchangeable, it is worth distinguishing three kinds at the outset. The first is biological stochasticity: the intrinsic randomness of the disease process itself, including transcriptional bursting, chromatin-state fluctuation, and stochastic phenotype switching. This is not a measurement artifact but a constitutive feature of the biology, and it is the source of much of the dynamic behavior this review is concerned with. The second is process noise in the formal sense: the way that biological stochasticity enters a dynamical model of the latent state, perturbing the trajectory of the system as it evolves. The third is measurement noise: the error introduced when the latent state is observed through an imperfect assay, and which corrupts the observation rather than the underlying biology. The distinction between the second and third is fundamental rather than semantic, because they enter at different stages—process noise perturbs the biology, whereas measurement noise perturbs only our view of it—and conflating them produces the category error of mistaking an assay fluctuation for a true biological transition. Section 2.2 formalizes process noise and measurement noise as distinct terms in the state and observation equations, respectively; biological stochasticity is the phenomenon that the process-noise term is intended to represent.

### 2.2 A mathematically explicit but restrained scaffold

The equations used in this review are not intended to define one universal model of cancer. Rather, they are representative formal motifs that make the biological argument more precise. We begin by representing the internal biological state of the tumor as a stochastic dynamical system:

$$\text{dX}\_\text{bio}\left(t\right)=f\left(X\_\text{bio}\left(t\right),u\left(t\right),t\right)\;dt+B\left(X\_\text{bio}\left(t\right),u\left(t\right),t\right)\;dW\_t$$ (1)

Here, X_bio denotes the latent biological state, f summarizes deterministic biological couplings, u represents intervention or external context, and B dW_t represents stochastic forcing (the process noise of the latent dynamics). For clarity, if $X\_\text{bio}\left(t\right)\in R^{n}$ and W_t is a q-dimensional standard Wiener process, then B $\left(X\_\text{bio}\left(t\right),u\left(t\right),t\right)$ is an n × q diffusion matrix. Writing the system in this way makes one assumption explicit: cancer evolution is partly deterministic and partly stochastic. Importantly, the stochastic term is allowed to be state dependent, which permits multiplicative-noise interpretations of transcriptional variability, state switching, and therapy-perturbed fluctuations, rather than reducing all uncertainty to an additive constant. Rare jump-like events may require richer noise models, but the Wiener form is sufficient for the formal scaffold used here. This equation resolves the ambiguity between deterministic progression and stochastic exploration that static biomarkers cannot distinguish. It assumes Markovian dynamics with state-dependent but continuous noise; validation would require densely sampled longitudinal molecular profiles, currently available only in model systems or selected liquid-biopsy cohorts.

Clinical and experimental observations belong to a linked but distinct layer:

$$L\left(t\right)=h\left(X\_\text{bio}\left(t\right),z\left(t\right),t\right)+\varepsilon \_obs\left(t\right)$$ (2)

$L\left(t\right)$ collects measured observables such as ctDNA, imaging, pathology, laboratory values, or symptoms; $z\left(t\right)$ denotes measurement context, and ε_obs denotes observation (measurement) noise. The crucial conceptual move is that L is not the disease itself. It is a projection of the latent biological state through imperfect assays, sparse sampling, and heterogeneous clinical context. That distinction protects biomarker interpretation from category errors; changes in ctDNA or imaging may reflect true state transitions, delayed observation of an earlier transition, or measurement artifact (Nguyen Phuong et al., 2025; Tie et al., 2022; Tie et al., 2025a). This equation makes explicit that all clinical assays involve an observation function with its own noise structure; its value is conceptual, preventing the category error of equating a biomarker change with a true biological transition. The main limitation is that h is typically unknown and must be estimated jointly with the latent state.

Near a local operating point X, Equation 1 can be linearized for interpretive purposes. If the diffusion term is locally frozen at $B=B\;\left(X,u,t*\right)$ with the same n×q dimensions as B in Equation 1, then the fluctuation dynamics obey:

$$d\delta X\left(t\right)\approx J\delta X\left(t\right)\;dt+B*\text{dW }\_t$$ (3)

where $\delta X=X\_\text{bio}-X*\text{ and }J=\partial f/\partial \left.X\right|\_\left\{X*\right\}$ . This is a local approximation, not a global theory of the disease. It supports two kinds of reasoning that are valuable in a review. First, local stability can be discussed through the spectrum of J: if all eigenvalues of J have strictly negative real parts, the operating point is locally asymptotically stable in the deterministic approximation. Second, the same local form explains why critical slowing down is expected near a loss of stability. As the dominant eigenvalue $\lambda_{1}$ approaches zero from below, recovery time increases approximately as $\tau \;∼\;{\left|\text{Re}\;\left(\lambda_{1}\right)\right|}^{-1}$ , variance in the corresponding mode grows, and lag-1 autocorrelation approaches one. These early-warning signatures were formalized for ecological and climate transitions by Scheffer et al. (2009), and extended methodologically by Dakos et al. (2012).

The point of Equation 3 is to connect state destabilization to measurable quantities—relaxation time, fluctuation amplitude, and autocorrelation—that can in principle be estimated from dense longitudinal data. Because observation is partial, model identifiability is a central limitation: structural identifiability (Villaverde et al., 2016) should precede parameter estimation, and practical identifiability should be assessed through profile likelihood or posterior geometry (Raue et al., 2009). Any cancer-dynamics program that ignores identifiability will overstate what can be inferred (Wiley et al., 2025; Fochesato et al., 2026).

Identifiability has a second face that is easily conflated with the first. Structural and practical identifiability concern whether model parameters can be recovered from data; causal identifiability concerns whether an interventional question—what would happen to the trajectory if a given layer were perturbed—can be answered at all from the data at hand. The two are distinct: a model can be parameter-identifiable yet causally uninformative, because longitudinal observation of a treated cohort confounds the effect of an intervention with the clinical reasons it was administered. In oncology specifically, treatment effects estimated from observational trajectories rest on an untestable no-unmeasured-confounding assumption, and no internal consistency check guarantees that this assumption holds (van Amsterdam et al., 2024). This matters acutely in the sparse, irregularly sampled, and actively perturbed setting that defines dynamic oncology, because the same data limitations that threaten parameter identifiability also obstruct causal identification. A method that estimates latent state well is not thereby licensed to identify causal leverage points or to recommend interventions; where the clinical question is genuinely interventional, the study design must support it—through randomization, a natural experiment, or causal assumptions encoded in advance—rather than relying on the predictive fit of a dynamical model. This distinction recurs in Section 3, where current learning-based methods are shown to be stronger at state estimation than at the causal and control questions that ultimately drive therapy.

BOX 1 **Core propositions of this review.**

- Cancer is better described as a dynamic, multiscale system rather than as a fixed molecular identity.
- Dynamic reasoning requires explicit distinction among state, transition, and trajectory.
- Noise is biologically constitutive, not merely a technical nuisance.
- Clinical measurements observe a projection of latent biology rather than the full disease state.
- Machine-learning methods are most valuable here when they improve inference under partial observability, not when they are treated as substitutes for mechanisms.
- Dynamic biomarkers become clinically meaningful only when linked to interventions that alter outcomes, not merely when they detect change.

## 3 Dynamic cancer data and computational inference

**FIGURE 2:**
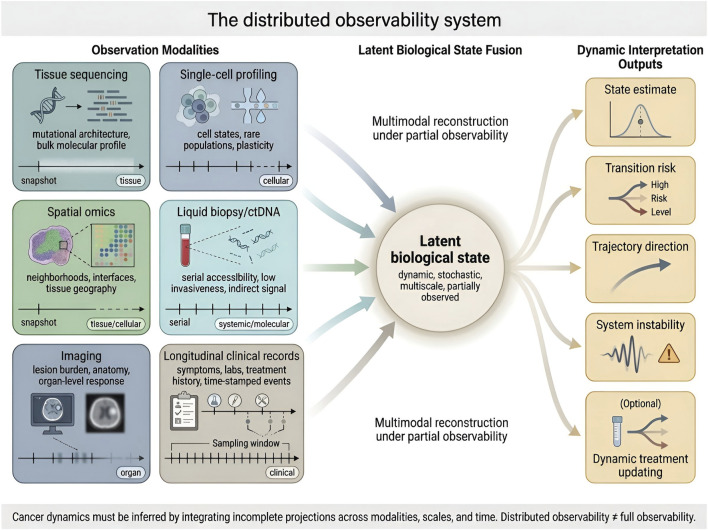
The distributed observability system. Schematic overview of the major data streams used to infer cancer dynamics: tissue sequencing, single-cell profiling, spatial omics, liquid biopsy, imaging, and longitudinal clinical records. No single modality spans all relevant scales. The central inference problem is therefore multimodal reconstruction of latent biological state under partial observability.

If cancer is dynamic, what data reveal those dynamics? No single modality spans all relevant scales. Tissue sequencing provides molecular detail but is sparse in time; single-cell profiling resolves heterogeneity but is destructive; spatial platforms such as Visium (Ståhl et al., 2016) and MERFISH (Chen et al., 2015) restore geography but remain largely static in routine use; liquid biopsy is serially accessible but indirect. Imaging covers lesions and organs but is biologically coarse. Multiplexed proteomic platforms, including proximity-extension assays and targeted mass spectrometry, are emerging as serially accessible molecular readouts complementary to transcriptomics, though clinical validation for longitudinal cancer monitoring lags behind ctDNA and imaging (Wenk et al., 2024). Electronic health records and routine laboratory data contribute longitudinal context but are noisy and inconsistently sampled. Dynamic oncology, therefore, operates as what might be called a distributed observability system: a collection of partial, asynchronous instruments that illuminate different shadows of the same evolving disease (Nguyen Phuong et al., 2025; Liu et al., 2026; Yuan, 2016). Figure 2 summarizes this distributed observability problem.

The term distributed observability is meant precisely, and three properties give it content. First, the instruments are heterogeneous in what they observe: in the language of Equation 2, each modality implements a different observation map onto a different subspace of the latent state—ctDNA projects onto tumor burden and clonal composition, imaging onto lesion size and texture, single-cell assays onto cellular composition—so no instrument is a lower-resolution version of another, and they cannot be ranked on a single axis of quality. Second, the instruments are asynchronous and non-commensurate in time: blood draws, scans, and biopsies are acquired on different schedules and at different frequencies, so the system rarely yields a coherent simultaneous snapshot of the latent state, and the gaps between observations are themselves informative about what can be inferred. Third, and consequently, the limiting problem is fusion rather than acquisition: because each instrument supplies only a partial projection, improving any single modality in isolation cannot reconstruct the trajectory, whereas jointly modeling several partial views can. This is what distinguishes distributed observability from a mere data-integration task. Integration presumes that the measured quantities are different features of the same accessible object; distributed observability recognizes that the object itself—the latent biological state of Equation 1—is never directly measured, and that every instrument returns a noisy, partial, time-shifted function of it. The practical implication is that the central question is not which assay is best but which combination of assays, sampled on which schedule, renders a given dynamic quantity identifiable at all.

This framing clarifies the role of computational modeling and machine learning. The central problem is not simply high-dimensional data integration—it is inference under partial observability. Each computational family addresses a different aspect of this inference problem, but none recovers the full disease dynamics. Latent-variable models such as scVI (Lopez et al., 2018) and multi-modal factor models such as MOFA+ (Argelaguet et al., 2020) compress and align measurements but do not encode temporal autocorrelation or intervention-driven nonstationarity. Velocity and trajectory methods (La Manno et al., 2018; Bergen et al., 2020; Lange et al., 2022; Saelens et al., 2019) provide local directional information but can be strained in therapy-perturbed cancer systems. Spatial graph models (Liu et al., 2026) and hybrid pharmacometric-learning approaches (Fochesato et al., 2026) capture spatial structure and longitudinal dose-response, respectively. The common limitation is that each method supplies constrained inference within its own observational window rather than mechanistic reconstruction of the underlying biological process.

Foundation models such as scGPT (Cui et al., 2024) and Geneformer (Theodoris et al., 2023) leverage self-supervised learning across millions of cells to build transferable representations. Their scale introduces useful priors for data-sparse settings, but temporal behavior remains weakly mechanistic and performance is sensitive to corpus composition and domain shift.

The distinction between state estimation and control matters. Current learning-based models are better at estimating latent state and forecasting short-horizon risk than at identifying causal leverage points, reflecting the partial observability and nonstationarity of cancer systems. The most useful benchmarks are therefore calibration, temporal generalization, perturbation robustness, and uncertainty quantification. Alternative families—including causal structure learning, switching state-space models, and mechanistic network inference—should be viewed as complementary, especially when the question concerns intervention or controllability. Where the latent state and its noise are modeled explicitly, classical state-estimation algorithms become directly applicable: Kalman and extended or unscented Kalman filters for approximately linear-Gaussian dynamics, particle filters (sequential Monte Carlo) for the nonlinear, non-Gaussian regimes typical of cancer, and Bayesian hierarchical models for pooling sparse, irregularly sampled longitudinal series across patients while propagating uncertainty. These methods instantiate the observation-and-state structure of Equations 1, 2, and their applicability is bounded by the same identifiability constraints discussed in Section 2.2. Table 1 summarizes representative computational tasks, typical use cases, and their main caveats in a dynamic oncology setting.

**TABLE 1:** Representative computational tasks for dynamic cancer inference.

Task	Representative methods	What they are useful for	Main caveat
Latent-state representation	scVI, variational latent-variable models (Lopez et al., 2018)	Compress noisy molecular measurements into lower-dimensional states while preserving uncertainty	Representation, not direct mechanistic causality
Multimodal integration	MOFA+, weighted-nearest-neighbor approaches, multimodal transformers (Argelaguet et al., 2020)	Align transcriptomic, epigenomic, proteomic, or imaging layers measured on partially overlapping samples	Mostly static factorization; temporal autocorrelation, regime shifts, and intervention effects are not usually explicit
Local directional inference	RNA velocity, scVelo, CellRank (La Manno et al., 2018; Bergen et al., 2020; Lange et al., 2022)	Estimate local transcriptional tendency, short-horizon state change, and probabilistic fate commitment	Steady-state or kinetic assumptions can fail under therapy, rapid perturbation, or CNV-heavy systems; direction remains local
Trajectory reconstruction	Slingshot, PAGA, Monocle 3, and related methods (Saelens et al., 2019)	Order cells along developmental or disease pseudotime trajectories	Sensitive to topology choice; no single method dominates across branching patterns; perturbation-aware trajectories remain challenging
Spatial structure extraction	Graph neural networks, niche-detection tools, spatial deconvolution (Liu et al., 2026; Yuan, 2016)	Map cellular neighborhoods, interfaces, and likely communication structure	Most datasets remain sparsely serial, so inferred change is often indirect
Longitudinal risk updating	Time-to-event models, state-space models, hybrid pharmacometric-learning approaches (Nguyen Phuong et al., 2025; Fochesato et al., 2026)	Integrate repeated blood, imaging, or clinical data to update recurrence or progression risk	Performance depends heavily on sampling design, missing-data structure, and how interventions are encoded
Foundation-model transfer	scGPT, Geneformer, and related large pretrained single-cell or multimodal models (Cui et al., 2024; Theodoris et al., 2023)	Support annotation, denoising, perturbation prediction, and hypothesis generation across large corpora	Broad priors can help, but temporal behavior remains weakly mechanistic and sensitive to corpus and domain shift
Signaling-entropy estimation	SCENT-like signaling-entropy approaches (Teschendorff and Enver, 2017)	Entropy-based proxies for regulatory flexibility or potency	Not direct estimators of attractor depth or transition barriers; should be interpreted as summary statistics of regulatory openness

Taken together, these methods are most persuasive when embedded in an explicit inferential question. Static multi-omics integration should be distinguished from dynamic trajectory alignment, and local directional inference from global barrier estimation or control.

A candid assessment of where these methods stand reveals an uneven landscape. At the clinically deployed end, variant-calling algorithms for ctDNA panels, MRD-detection thresholds in hematologic cancers, and guideline-concordance checks in clinical decision-support systems represent AI applications that have entered routine practice—though even here, standardization and equity challenges persist (Wang et al., 2023; Febbo et al., 2024). At the emerging-but-validated end, latent-variable models such as scVI and trajectory-inference tools such as CellRank have been rigorously benchmarked on curated datasets, though their performance under treatment-perturbed, longitudinally sampled cancer data remains less well characterized (Luecken et al., 2022; Lange et al., 2022). At the speculative end, foundation models for single-cell biology—notably scGPT and Geneformer—have attracted significant attention, but recent zero-shot evaluations have shown that these models can underperform simpler baselines such as highly-variable-gene selection combined with scVI or Harmony on standard clustering tasks, and that their temporal generalization properties remain untested (Kedzierska et al., 2025; Cui et al., 2024; Theodoris et al., 2023). Neural ODE and SDE models for patient-trajectory prediction and reinforcement-learning approaches for adaptive therapy remain theoretical proofs of concept without clinical deployment. This stratification matters for the framework proposed here because the most mature AI tools support observation—signal detection, variant calling, MRD quantification—rather than the latent-state estimation or transition-risk inference that a dynamic oncology program ultimately requires. Bridging this gap will demand benchmarks that go beyond annotation accuracy to test temporal coherence, perturbation robustness, calibrated uncertainty quantification, and whether the AI-derived signal changes a therapeutic decision.

## 4 Six internal biological layers of cancer dynamics

The six layers below are analytically separable but biologically coupled. They should be read as recurring dynamic scales rather than independent compartments. Each subsection introduces a layer-specific formal motif, not because any single equation captures the full complexity of the layer, but because explicit motifs make the assumptions, variables, and uncertainties of each layer easier to discuss. The six layers differ substantially in evidentiary maturity. Molecular regulatory dynamics (Section 4.1) and cellular state plasticity (Section 4.2) are supported by extensive single-cell experimental evidence across multiple cancer types and are approaching prospective clinical application. Spatial organization (Section 4.3) and tumor ecosystem co-evolution (Section 4.4) rest on growing spatial-omics and evolutionary-oncology literatures but remain largely retrospective. Metabolic-epigenetic coupling (Section 4.5) and mechanobiological feedback (Section 4.6) are the most conceptually motivated but empirically earliest layers; their inclusion reflects their coupling importance rather than a claim of equivalent clinical maturity.

### 4.1 Molecular regulatory dynamics

At the molecular scale, the core dynamic problem is not only which mutations or pathways are present, but how regulatory circuits stabilize or destabilize phenotypic programs through time. Cancer cells often occupy semi-stable transcriptional configurations maintained by feedback among transcription factors, chromatin regulators, signaling nodes, and stress responses. In this context, attractor language is useful because it emphasizes occupancy and accessibility, not merely abundance. The Waddington landscape metaphor, formalized quantitatively by Huang et al. (2009) and by Wang et al. (2011), frames cell states as basins of attraction in a high-dimensional regulatory space, with transitions governed by the depth and geometry of those basins. A therapy may leave a mutation unchanged but still alter the depth or accessibility of a regulatory state by rewiring feedback and noise (Tirosh and Suva, 2024; Goldberg et al., 2007).

$$dx_{i}/dt=g_{i}\left(x,m,t\right)-\gamma_{i}x_{i}+\sigma_{i}\left(x,t\right)\xi_{i}\left(t\right)$$ (4)

Equation 4 is a generic motif for a noisy gene-regulatory node: production $g_{i}$ depends on the state of the network x and external or metabolic context m; degradation occurs at rate $\gamma_{i}$ ; and noise enters through $\sigma_{i}\left(x,t\right)\xi_{i}\left(t\right)$ , where $\xi_{i}\left(t\right)$ is a white-noise process (the informal Langevin representation of the Wiener increment used in Equation 1). The biological value of writing, even this simple form, is that it makes explicit why molecular biomarkers are insufficient when interpreted as static labels. A mutation panel describes part of the network architecture; it does not report the current occupancy of a regulatory state, the ease of transition to a neighboring state, or the effect of therapy on fluctuation amplitude. This is one reason single-cell studies increasingly emphasize programs, modules, and regulatory topology rather than isolated markers (Hanahan, 2022; Tirosh and Suva, 2024).

Noise is especially important here: transcriptional bursting and epigenetic switching can make a state appear heterogeneous even when its average profile is stable, while low-noise states may look homogeneous yet retain potential for abrupt transition. Molecular dynamics thus sets the stage for all downstream layers (Gupta et al., 2011; Mojtahedi et al., 2016).

### 4.2 Cellular state plasticity

The cellular layer is where dynamics become phenotypically legible. Single-cell studies now show that malignant populations are rarely composed of one fixed identity. Instead, they occupy mixtures of proliferative, stressed, invasive, persister-like, stem-like, or therapy-adapted states that can interconvert on experimentally relevant timescales. The critical biological issue is therefore not only heterogeneity, but plasticity: how often cells move, how reversible those movements are, and what constraints make some trajectories more likely than others (Tirosh and Suva, 2024; Gupta et al., 2011).

$${dp}_{i}/\text{dt}=\Sigma \_\left\{j\neq i\right\}\left(k\_\left\{ji\right\}p_{j}-k\_\left\{ij\right\}p_{i}\right)+p_{i}\left(r_{i}-\bar{r}\right)$$ (5)

Equation 5 is a useful motif for state plasticity, but it should be read as a mean-field population-balance approximation. Here, $p_{i}$ is the fraction of cells in state i ; $k_{ji}p_{j}$ denotes influx from state j to state i, $k_{ij}p_{i}$ denotes outflux from state i to state j , $r_{i}$ is the growth or persistence advantage of state i, and $\bar{r}$ is the population-average fitness. The first term captures stochastic switching in expectation. The second captures selection. This equation clarifies why noise and therapy matter simultaneously: fluctuations create phenotypic exploration, while selection amplifies some explored states and suppresses others. In finite populations, demographic and state-switching noise can drive rare but clinically important states to be entered or exited through fluctuations even when the mean-field drift is small—a principle with direct relevance to drug-tolerant persistent emergence and minimal residual disease (Sharma et al., 2010; Hangauer et al., 2017). A minimal stochastic analogue would write $d_{p}=F\left(p,t\right)\;dt+G\left(p,t\right)\text{dW}\_t$ , where G collects demographic and state-switching noise; the exact form is application-specific and usually not identifiable from sparse clinical sampling alone.

Drug tolerance provides an important test: Sharma et al. (2010) showed that persister states arise reversibly through chromatin-mediated mechanisms, and Hangauer et al. (2017) demonstrated that these persisters acquire targetable GPX4 dependence—illustrating that phenotypic exploration under therapy follows constrained regulatory paths captured by Equation 5.

Velocity-based methods have made this layer especially visible, but require careful interpretation. RNA velocity (La Manno et al., 2018), its dynamical extension scVelo (Bergen et al., 2020), and the probabilistic fate-estimation framework CellRank (Lange et al., 2022) provide local to intermediate-range dynamical information, but do not estimate transition-barrier heights with certainty and may be violated in therapy-exposed or strongly branching cancer systems. Lineage-tracing technologies (Weinreb et al., 2020; Rodriguez-Fraticelli et al., 2018) now offer direct fate measurement that can calibrate computational models.

Longitudinal studies reinforce this point: the TRACERx program demonstrated branching evolution and spatially structured selection in lung cancer (Jamal-Hanjani et al., 2017), with ctDNA analyses showing that circulating measurements can track some but not all of these transitions (Abbosh et al., 2017).

### 4.3 Spatial organization and niche dynamics

Cancer dynamics do not occur in a spatial vacuum. Tissue geography organizes gradients of oxygen, metabolites, immune pressure, stromal density, and drug exposure. As a result, the same molecular or cellular state can behave differently depending on where it sits. Spatial heterogeneity is therefore not only a descriptive detail; it changes the transition structure of the disease. This is why spatially resolved studies have become indispensable for understanding immune exclusion, niche-specific adaptation, and compartmentalized treatment response (Liu et al., 2026; Yuan, 2016).

$$\partial u/\partial t=D\;\nabla^{2}u+F\left(u,v,x,t\right)$$ (6)

Equation 6 represents a general spatial motif in which a field u—such as nutrient concentration, immune activity, or cell density—changes through diffusion, transport, and local reaction F; here v denotes other interacting field variables such as stromal or immune densities, and x denotes spatial position. This is not meant to imply that all tumor geography is diffusion-driven. Rather, it captures the key systems insight that spatial organization changes because local processes and transport are jointly operating. Reaction-diffusion models (Anderson and Quaranta, 2008) and agent-based simulations (Macklin et al., 2012; Metzcar et al., 2019) encode that principle. Cell-based computational models are inherently stochastic and simulate individual cells as they interact in virtual tissues, making them a natural complement to the continuum motifs used in this article, each trading spatial resolution against computational tractability.

Spatial transcriptomics now permits direct observation of niche structure. Platforms ranging from spatially barcoded approaches such as Visium (Ståhl et al., 2016) to single-molecule FISH methods such as MERFISH (Chen et al., 2015) have revealed reproducible spatial niches in tumors, including immune-excluded borders, hypoxic cores, and spatially confined stem-like compartments (Liu et al., 2026). However, spatial data remain mostly static, and spatial inference should be interpreted as one window into state and coupling rather than a complete record of the trajectory (Liu et al., 2026).

### 4.4 Tumor ecosystem co-evolution

At the ecosystem scale, progression emerges from interactions among malignant cells, immune cells, fibroblasts, vasculature, extracellular matrix, and soluble mediators. This is the layer in which ecological and evolutionary reasoning becomes most directly useful. Therapy does not act on a homogeneous tumor mass—it perturbs a competitive and cooperative ecosystem in which sensitive and resistant populations, immune effectors and suppressors, and stromal constraints all reshape one another through time (Merlo et al., 2006; Gatenby and Brown, 2020).

$${dN}_{i}/\text{dt}=N_{i}\left(r_{i}+\Sigma_{j}a_{\text{ij}}N_{j}\right)$$ (7)

Equation 7 is a generalized ecological motif in which $N_{i}$ denotes population abundance and $a\_\left\{ij\right\}$ encodes competitive, cooperative, or predatory interactions. As written, the motif is a mean-field approximation. When spatial structure matters, a minimal extension is ${{\partial N}_{i}/\partial_{t}=D_{i}\;\nabla^{2}N}_{i}+N_{i}\left(r_{i}+\Sigma_{j}a_{\text{ij}}N_{j}\right)$ , where $D_{i}$ represents dispersal or local mixing. The reason this motif matters clinically is that treatment also changes the interaction matrix, not just the abundance of one compartment. A drug that strongly suppresses sensitive cells may inadvertently release resistant cells from competition; an immunotherapy that alters one ecological bottleneck may fail if another layer is dominant.

Immune dynamics deserve particular attention here. The tumor–immune interaction is a dynamic co-evolutionary process in which T cell clones expand, contract, and undergo exhaustion on timescales that interact with tumor evolution (Chen and Mellman, 2013; Yost et al., 2019). Spatial analyses show that immune exclusion is not a fixed state but an evolving boundary shaped by stromal remodeling and mechanical access (Joyce and Fearon, 2015). In the language of Equation 7, immune populations are not external forces but endogenous ecological partners whose interaction coefficients change with immunotherapy, radiation, and even mechanical remodeling of the tumor microenvironment.

Adaptive therapy provides the clearest clinical example of ecosystem-aware intervention. Gatenby et al. (2009) proposed modulating treatment intensity to preserve sensitive competitors, and Zhang et al. (2017), Zhang et al. (2022) translated this into adaptive abiraterone treatment for mCRPC, showing that evolutionary reasoning could prolong response. Enriquez-Navas et al. (2016) extended the concept preclinically to breast cancer. No phase III trial has been completed, and the mCRPC studies remain single-arm, but these studies demonstrate that ecological motifs can inform intervention design.

### 4.5 Metabolic-epigenetic coupling and dormancy

Metabolism is often summarized as a hallmark of cancer, but its dynamic role is broader than fuel provisioning. This layer currently rests on biochemical plausibility and retrospective associations rather than on prospective longitudinal validation; it is included because metabolic-epigenetic coupling provides a mechanistic bridge between the molecular and dormancy phenomena discussed elsewhere, not because it has yet achieved the evidentiary depth of the molecular or cellular layers. Metabolic state determines cofactor availability, redox buffering, stress tolerance, and chromatin accessibility; in turn, chromatin state regulates which metabolic programs can be deployed. This bidirectional coupling matters because many clinically important transitions—drug persistence, quiescence, lineage infidelity, immune escape, and metastatic dormancy—depend on metabolic feasibility as much as on transcriptional possibility (Pavlova and Thompson, 2016; Kaelin and McKnight, 2013; Dai et al., 2020).

The mechanistic basis for this coupling is now concrete rather than conjectural, even where its dynamic role remains under-characterized. Several chromatin-modifying enzymes are obligate consumers of central-carbon metabolites, so their activity tracks metabolic state directly: the α-ketoglutarate-dependent TET DNA hydroxylases and Jumonji-family histone demethylases require α-ketoglutarate as a co-substrate, histone acetyltransferases require acetyl-CoA, and methyltransferases require S-adenosylmethionine. The clearest demonstration comes from isocitrate-dehydrogenase-mutant cancers, in which the oncometabolite 2-hydroxyglutarate accumulates to millimolar levels and competitively inhibits these α-ketoglutarate-dependent dioxygenases, producing genome-wide increases in histone and DNA methylation and a block in differentiation (Xu et al., 2011). This is a direct, structurally resolved instance of a metabolic perturbation rewiring the epigenome, and it establishes the general principle—fluctuations in metabolite pools are read out as changes in chromatin state, and therefore in which transcriptional programs are accessible. Reciprocally, chromatin state gates the expression of metabolic enzymes and transporters, closing the loop. What remains immature is not the existence of these mechanisms but their longitudinal, patient-level quantification: cofactor pools, local enzyme kinetics, and chromatin occupancy are rarely measured together over time in the same tumor, so the coupling is well documented at the level of mechanism yet largely unparameterized at the level of dynamics (Pavlova and Thompson, 2016; Dai et al., 2020).

$$dm/\text{dt}=F\_m\left(m,c,e,t\right),\text{dc}/\text{dt}=F\_c\left(c,m,t\right),\text{dq}/\text{dt}=F\_q\left(q,m,c,n,t\right)$$ (8)

Equation 8 is a compact motif: m denotes metabolic state, c chromatin state, and q dormancy propensity. Dormancy emerges from coupled processes in which metabolite availability changes chromatin regulators and niche signals reinforce quiescence or awakening. Kaelin and McKnight (2013) established the biochemical basis for metabolic regulation of chromatin state through cofactor availability. Dai et al. (2020) extended this into a broader chromatin-metabolism framework. This motif is the most schematic of the six; no single-patient parameterization has been achieved. Its value is organizational: it makes explicit why dormancy cannot be modeled as an intrinsic cell property without metabolic and niche inputs.

Dormancy is the sharpest test of this layer. Dormant cells occupy a persistence program shaped by stress signaling, epigenetic repression, and metabolic restraint (Aguirre-Ghiso, 2007; Sosa et al., 2014; Sosa et al., 2015), with regulatory architecture overlapping drug-tolerant persister states (Section 4.2).

### 4.6 Mechanobiological feedback

Mechanobiology is the layer with the strongest biophysical rationale but the least direct clinical-oncology evidence among the six layers presented here. Its inclusion reflects its coupling importance—particularly to the spatial, ecological, and observability layers—rather than a claim of equivalent translational maturity. Matrix stiffness, solid stress, interstitial pressure, confinement, and shear forces are not merely contextual background; they compress vessels, reduce perfusion, worsen drug delivery, intensify hypoxia, alter cell shape and adhesion, and can stabilize particular phenotypes over time (Butcher et al., 2009; Lu et al., 2012; Jain et al., 2014).

Two findings anchor this layer mechanistically. First, matrix rigidity is not merely permissive but instructive: in mammary epithelium, increasing extracellular-matrix stiffness clusters integrins, elevates Rho-dependent cytoskeletal tension and focal-adhesion signaling, disrupts tissue architecture, and drives a malignant phenotype, while lowering tension or contractility can phenotypically revert transformed cells—establishing tissue mechanics as a regulator of cell fate rather than a passive consequence of it (Paszek et al., 2005). Second, the downstream transcriptional route is now identified: the co-activators YAP and TAZ act as nuclear relays of mechanical input, translating ECM stiffness and cell geometry into transcriptional programs in a manner dependent on Rho activity and actomyosin tension but largely independent of the canonical Hippo kinase cascade (Dupont et al., 2011). Together these results convert the abstract notion of mechanical feedback into a concrete circuit—stiffness and stress are sensed at adhesions, transduced through cytoskeletal tension, and read out as YAP/TAZ-dependent transcription, which in turn alters matrix deposition and contractility and thus future stiffness. This is the biological content behind the reciprocal-reinforcement motif formalized below. As with the metabolic layer, the limiting gap is dynamic and clinical rather than mechanistic: these circuits are characterized largely in engineered substrates and cell systems, and their parameters have not been measured longitudinally in patient tumors.

$$\text{dY}/\text{dt}=\alpha \;S/\left(K+S\right)-\beta Y,\text{dS}/\text{dt}=G\left(\text{ECM},N,Y\right)-\lambda S$$ (9)

Equation 9 gives one useful motif for mechanobiological feedback. Y can be read as activity of mechanotransduction outputs such as YAP/TAZ , while S represents effective mechanical stress or stiffness. Mechanical load activates YAP/TAZ -like programs, and those programs, in turn, alter extracellular matrix deposition, contractility, invasion, and thus future stress. The loop is not universal in parameterization, but it captures the key systems principle of reciprocal reinforcement. If feedback from Y into stress generation is sufficiently strong relative to decay, the nullclines can intersect in a way that yields bistability or hysteresis between low-stress/low-Y and high-stress/high-Y regimes, making mechanobiology a plausible contributor to switch-like phenotypic persistence. The main limitation is that the parameters in this motif are currently estimable only from *in vitro* mechanobiology experiments such as hydrogel systems and AFM-calibrated substrates, not from clinical tumor data; translation to patient-level inference would require coupling to imaging-based stiffness surrogates such as MR elastography. YAP/TAZ mechanoregulation has been characterized across physiological and pathological contexts (Cai et al., 2021; Piccolo et al., 2023), but dynamic parameterization in living tumors remains out of reach.

Mechanobiology also matters for observability: a biomarker measured in blood may reflect whether compressive stress has altered perfusion or whether fibrosis has created an immune-excluded niche. Mechanical conditions couple strongly to spatial, ecological, and observation layers (Jain et al., 2014; Lu et al., 2012).

## 5 A unified representation of coupled cancer dynamics

**FIGURE 3:**
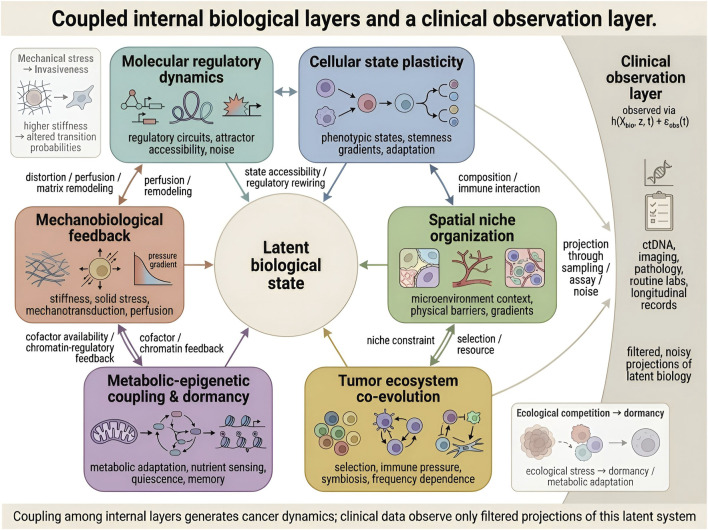
Coupled internal biological layers and a clinical observation layer. Diagram showing the six internal layers discussed in Section 4 and the distinct longitudinal clinical layer discussed in Section 6. Arrows depict recurring coupling motifs linking molecular, cellular, spatial, ecological, metabolic-dormancy, and mechanobiological dynamics, together with their projection into clinical observables. Example callouts illustrate mechanobiology-to-plasticity and ecology-to-dormancy couplings.

The six internal layers above can be represented collectively as a latent biological state vector:

$$X\_\text{bio}\left(t\right)={\left[M\left(t\right),C\left(t\right),Sp\left(t\right),E\left(t\right),D\left(t\right),Mec\left(t\right)\right]}^{T}$$ (10)

where M,C,Sp,E,D , and Mec denote molecular, cellular, spatial, ecological, metabolic-dormancy, and mechanobiological states, respectively (here Sp denotes the spatial layer, distinct from the mechanical stress S in Equation 9). The longitudinal clinical layer remains distinct and is represented through the observation map in Equation 2. This separation is deliberate. It prevents a common conceptual error in biomarker science: treating the measured signal as if it were the disease itself. In reality, ctDNA, radiographic change, pathology, and routine laboratory values are conditional observations of the latent biological system, filtered through assay properties, sampling schedules, anatomy, and clinical context (Nguyen Phuong et al., 2025; Tie et al., 2022; Holder et al., 2024). The architecture and representative couplings are summarized in Figure 3.

This six-plus-one architecture also clarifies what cross-layer coupling means in practice. Several coupling motifs recur: regulatory rewiring changes which cellular states are accessible; spatial architecture channels ecological interaction; ecological competition reshapes metabolic demand and dormancy; mechanical forces alter spatial organization and ecological access; and all six layers distort the clinical signals through which the disease is monitored. These are not universal laws; they are recurring coupling patterns that make dynamic biomarkers difficult to interpret when isolated from biology (Wiley et al., 2025; Yuan, 2016; Jain et al., 2014; Aguirre-Ghiso, 2007).

To make that structure explicit, the local Jacobian can be written as a block matrix whose diagonal terms encode within-layer dynamics and whose off-diagonal terms encode cross-layer coupling. Two illustrative couplings are instructive: mechanobiology can modulate cellular plasticity by making transition rates stress dependent, and the metabolic-dormancy state can feed back to ecological interaction by altering the competitive interaction matrix itself.

**FIGURE 4:**
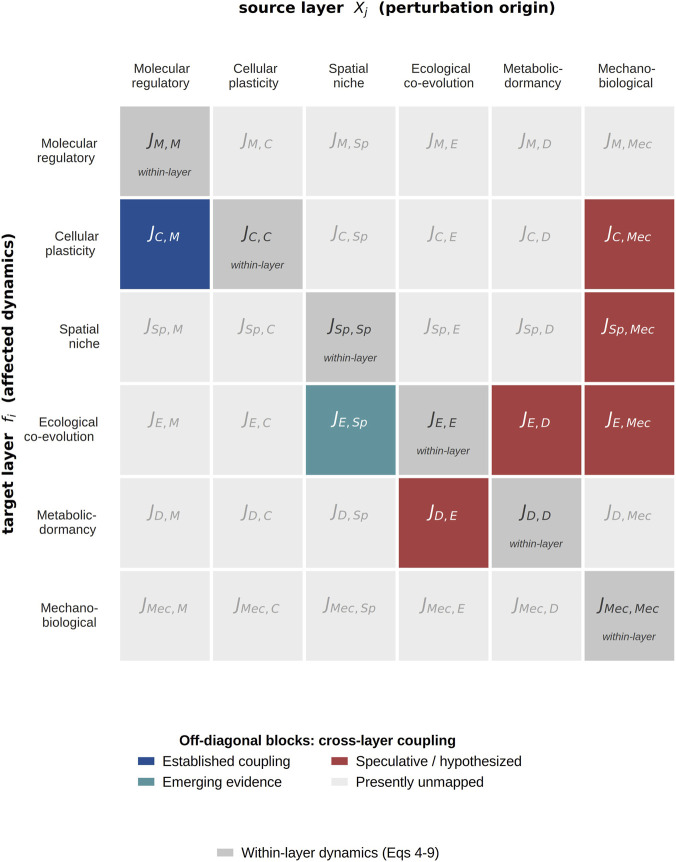
The block Jacobian of the six-layer system, annotated by evidentiary status. Local linearization of the composite latent state (Equation 10) about an operating point yields a 6 × 6 block matrix $J=\left[J\_\text{ab}\right]$ (Equation 11). Diagonal blocks (gray) are the within-layer dynamics formalized by the layer-specific motifs in Equations 4–9. Off-diagonal blocks J_ab=∂f_a/∂X_b represent the sensitivity of target layer a (rows) to perturbations originating in source layer b (columns). Each candidate coupling is colored at the evidentiary tier of its least-characterized endpoint layer, following the layer-maturity ranking in Section 4: established $\left(J\_\text{CM}\right)$ , emerging $\left(J\_E,\text{Sp}\right)$ , or speculative $\left(J\_D,E,J\_\text{Sp},\text{Mec},J\_E,\text{Mec},J\_C,\text{Mec},J\_E,D\right)$ . All remaining off-diagonal blocks are presently unmapped. The sparsity of the matrix is deliberate and substantive: most cross-layer couplings are not yet quantifiable, which is the central reason dynamic biomarkers are difficult to interpret in isolation from the layers that generate them.

To make this structure concrete rather than rhetorical, we write the local block Jacobian of the six-layer system explicitly. Linearizing the internal dynamics of Equation 10 about a local operating point X* yields a 6 × 6 block matrix

$$J=\left[\;J\_\text{ab }\right],a,b\in \left\{M,C,\text{Sp},E,D,\text{Mec}\right\}$$ (11)

in which each diagonal block J_aa is the within-layer dynamics already introduced as the layer-specific motifs (Equations 4–9), and each off-diagonal block J_ab=∂f_a/∂X_b encodes the sensitivity of layer a to perturbations originating in layer b. Writing the matrix this way forces an honest accounting: most off-diagonal blocks are not currently estimable, and the few that the present literature supports differ sharply in evidentiary strength. Figure 4 annotates each block by that strength. Following the layer-maturity ranking set out at the start of Section 4, we assign each candidate coupling the evidentiary tier of its least-characterized endpoint layer. On that basis, only the molecular-to-cellular coupling J_CM rests on established gene-regulatory and single-cell evidence—here the tier reflects not merely the maturity of the two endpoint layers but direct evidence for the coupling itself, in which regulatory-network state shapes the accessibility and depth of cellular attractor states and thereby drives cell-state transitions (Tirosh and Suva, 2024; Goldberg et al., 2007); the spatial-to-ecological coupling J_E,Sp reflects an emerging but largely retrospective spatial-omics literature; and every coupling that touches the metabolic-dormancy or mechanobiological layers— J_D,E,J_Sp,Mec,J_E,Mec,J_C,Mec , and J_E,D —remains hypothesized, awaiting longitudinal parameterization. All remaining off-diagonal blocks are presently unmapped.

This explicit but sparsely populated matrix is itself the central point. The preponderance of unmapped blocks is not a deficiency of the figure; it is an accurate depiction of the field. It is precisely because cross-layer couplings remain so weakly characterized that dynamic biomarkers are difficult to interpret when read in isolation from the layers that generate them. The block structure also makes the alternative reading explicit: where an off-diagonal block is non-negligible, a transition detected in one layer cannot be assumed to remain confined to it.

These examples are illustrative; full parameterization of all off-diagonal couplings across the six layers for a given tumor type is presently infeasible, and in practice coupling inference will require reduced-order models, targeted perturbation experiments, and layer-specific priors. The value of this composite representation is therefore as an organizing scaffold: it specifies what a complete dynamic model would need to contain and thereby clarifies what current models are leaving out.

Near a local operating point, Equation 10 inherits the local linearization logic of Equation 3, allowing cautious discussion of local stability. Because the disease is noisy, partially observed, and actively perturbed, local stability should be interpreted as one diagnostic property of the model rather than a direct clinical readout. Strong off-diagonal blocks indicate that destabilization in one layer is unlikely to remain confined to that layer.

For convenience, Table 2 consolidates the eleven formal motifs introduced across Sections 2, 4, 5, mapping each to the biological meaning it encodes, the kind of data that would be required to estimate or validate it, and its principal limitation in a realistic oncology setting.

**TABLE 2:** Summary of formal motifs: biological meaning, data requirements, and limitations.

Motif	Biological meaning	Data needed to estimate/validate	Principal limitation
Equation 1	Latent biological state evolves as a stochastic dynamical system: deterministic couplings plus state-dependent process noise	Densely sampled longitudinal molecular profiles (model systems or selected liquid-biopsy cohorts)	Assumes Markovian dynamics with continuous noise; rare jump-like events need richer models
Equation 2	Clinical observation is a noisy, partial projection of the latent state through an assay-specific observation map	Paired latent-state and assay measurements to estimate the observation function	The observation map h is typically unknown and must be estimated jointly with the latent state
Equation 3	Local linearization: fluctuations near an operating point are governed by the Jacobian, enabling stability and critical-slowing-down reasoning	Dense longitudinal data to estimate relaxation time, fluctuation amplitude, and lag-1 autocorrelation	A local approximation only; identifiability must be checked before parameters are trusted
Equation 4	Noisy gene-regulatory node: production, degradation, and intrinsic transcriptional noise at the molecular layer	Single-cell time-series at resolution sufficient to fit gene-specific noise parameters (largely reporter systems)	Assumes mean-field noise, fixed topology, and time-invariant degradation; therapy reshapes topology
Equation 5	Cellular-state plasticity as a mean-field population balance of stochastic switching plus selection	Lineage-tracing (expressed barcodes, Cas9 recorders), currently limited to model systems	Assumes transition rates are stationary between measurements, violated by intermittent therapy
Equation 6	Spatial niche dynamics: a reaction-diffusion field coupling local processes and transport	Time-resolved spatial profiling (serial biopsies with matched spatial transcriptomics)	Continuum approximation averages discrete cell-level events; serial spatial data remain scarce
Equation 7	Tumor-ecosystem co-evolution: generalized Lotka-Volterra interactions among malignant, immune, and stromal populations	Longitudinal clonal-frequency data from liquid biopsy or multi-region sequencing	Parameterizing the interaction matrix from patient data is the principal bottleneck
Equation 8	Metabolic-epigenetic-dormancy coupling: metabolite, chromatin, and dormancy states evolve jointly	Co-measured cofactor pools, chromatin occupancy, and niche signals over time in the same tumor	Most schematic of the motifs; no single-patient parameterization has been achieved
Equation 9	Mechanobiological feedback: mechanotransduction output and effective stress reinforce one another, permitting bistability	*In vitro* mechanobiology (hydrogels, AFM); clinically, imaging-based stiffness surrogates such as MR elastography	Parameters estimable only *in vitro* at present, not from clinical tumor data
Equation 10	Composite latent state: the six internal layers assembled into a single biological state vector, distinct from clinical observation	Joint, multi-layer longitudinal measurement of all six layers in one tumor type	Full parameterization across all six layers for a given tumor is presently infeasible
Equation 11	Block Jacobian: within-layer dynamics on the diagonal, cross-layer couplings off-diagonal, annotated by evidentiary status	Targeted perturbation experiments and reduced-order models with layer-specific priors	Most off-diagonal couplings are presently unmapped; full specification is infeasible (see Figure 4)

## 6 Dynamic biomarkers, adaptive oncology, regulation, and equity

Once cancer is framed as a dynamic and partially observed system, the biomarker problem changes. A biomarker is no longer just a variable associated with prognosis or treatment response at one time point. It can also be a signal of motion: a change in ctDNA burden, instability of a transcriptional state, emergence of a resistant subclone, or failure of a spatially localized response to generalize across the lesion. Dynamic biomarkers are therefore valuable not because they are measured serially *per se*, but because their meaning depends on time, direction, and context (Passaro et al., 2024; Holder et al., 2024).

**FIGURE 5:**
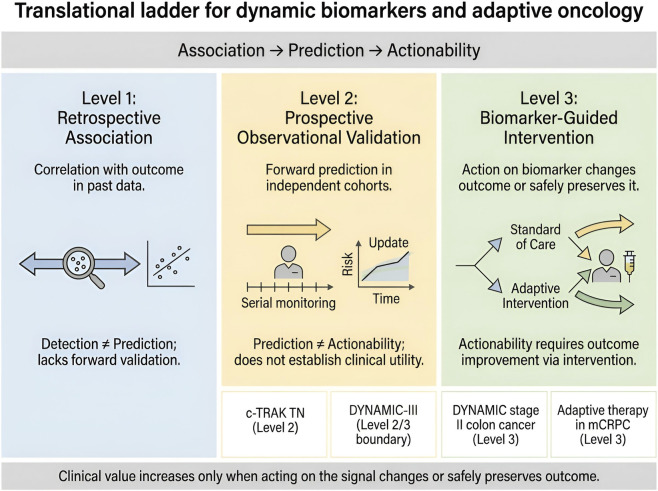
Translational ladder for dynamic biomarkers and adaptive oncology. Three-level framework for evaluating dynamic biomarkers. Level 1, retrospective association; Level 2, prospective observational validation; Level 3, biomarker-guided intervention. Representative examples include c-TRAK TN, the DYNAMIC-III program, the DYNAMIC stage II colon-cancer trial, MRD-guided therapy in hematologic malignancies, and adaptive-therapy studies in metastatic prostate cancer.

A useful way to organize the translational landscape is a three-level evidence ladder: Level 1 (retrospective longitudinal associations), Level 2 (prospective observational validation), and Level 3 (biomarker-guided intervention trials in which acting on the signal changes management and improves outcomes). This ladder separates detecting a dynamic signal from proving that it is actionable. Figure 5 and Table 3 summarize this framework.

**TABLE 3:** A practical evidence ladder for dynamic biomarkers.

Evidence level	Definition	Representative example	Interpretive implication
Level 1	Retrospective or secondary longitudinal association	Serial ctDNA, CTC counts, or delta-radiomic features associated with recurrence or survival	Demonstrates correlation with outcome, not yet actionability
Level 2	Prospective observational validation	Prospectively collected biomarker dynamics predict future course without dictating therapy	Demonstrates forward prediction, but does not benefit from acting on the signal
Level 3	Biomarker-guided intervention	ctDNA-guided adjuvant treatment in stage II colon cancer (DYNAMIC); adaptive abiraterone strategies in mCRPC; MRD-guided therapy in hematologic malignancies	Tests whether acting on the dynamic signal changes or safely preserves the outcome

The ladder is not merely taxonomic; its utility lies in explaining why some biomarkers stall between levels. Two instructive examples illustrate this. First, tumor mutational burden (TMB) was granted tumor-agnostic FDA approval at a threshold of 10 mutations per megabase as a companion diagnostic for pembrolizumab, but subsequent analyses across cancer types have shown that high TMB does not consistently predict immunotherapy response—particularly in breast, prostate, and adrenocortical cancers—owing to variability in mutation quality, assay platforms, and the biological heterogeneity of what high TMB captures (Niknafs et al., 2025; Ahmed et al., 2023). TMB thus sits uncomfortably between Levels 2 and 3: FDA-approved yet inconsistently actionable. Second, circulating tumor cell enumeration via CellSearch demonstrated robust Level 1–2 prognostic value across metastatic breast, prostate, and colorectal cancers (Section 6.2), but the SWOG S0500 trial found that switching chemotherapy based on persistently elevated CTCs did not improve survival, representing a concrete Level 3 failure (Smerage et al., 2014). These examples underscore a recurring pattern: a biomarker that detects a biologically real phenomenon may still fail at Level 3 if the triggered intervention does not match the biological layer driving the detected transition.

### 6.1 Circulating tumor DNA

The DYNAMIC stage II colon-cancer trial remains the clearest current example of Level 3 evidence in ctDNA-guided adjuvant therapy. A ctDNA-guided strategy reduced chemotherapy use without compromising 2-year recurrence-free survival, and 5-year follow-up supported the durability of that approach (Tie et al., 2022; Tie et al., 2025a). By contrast, the randomized phase 2/3 DYNAMIC-III study in stage III disease is more nuanced. It showed that ctDNA-guided de-escalation reduced oxaliplatin exposure and hospitalizations, but formal non-inferiority for the primary de-escalation analysis was not established overall, even though subgroup results in clinically low-risk tumors were encouraging (Tie et al., 2025b). This distinction matters: dynamic biomarkers can be clinically informative without yet being universally practice-changing.

The c-TRAK TN study makes a second critical point. Signal detection alone does not guarantee that the triggered intervention will improve the outcome. In c-TRAK TN, ctDNA detection identified a population at very high risk, but many ctDNA-positive patients already had radiologically evident metastatic disease by the time the trigger was acted on, and the ctDNA-positive population was biologically heterogeneous rather than uniformly immunotherapy-responsive (Turner et al., 2023). In the language of this review, the layer driving the detected transition matters. A biomarker can be dynamically valid yet mechanistically mismatched to the intervention chosen in response. An additional consideration is the distinction between tumor-informed and tumor-naïve assay strategies, which trade sensitivity against applicability (Corcoran and Chabner, 2018).

### 6.2 Circulating tumor cells

Circulating tumor cells (CTCs) provide a complementary readout, preserving intact cell-level information including morphology and transcriptional state. CellSearch-based enumeration established CTC counts as prognostic in metastatic breast, prostate, and colorectal cancers (Cristofanilli et al., 2004; de Bono et al., 2008; Cohen et al., 2008), and single-cell CTC profiling has revealed phenotypic heterogeneity including clusters with enhanced metastatic potential (Aceto et al., 2014). Serial CTC monitoring offers a window into the evolving circulating compartment, though its relationship to the full latent tumor state remains partially understood.

### 6.3 Serial imaging and delta-radiomics

Imaging-based dynamic biomarkers operate at the organ and lesion scale. Standard response criteria such as RECIST do not capture within-lesion heterogeneity or treatment-induced texture changes. Delta-radiomic approaches—in which quantitative imaging features are extracted at multiple time points and their change trajectories analyzed—offer richer dynamic readouts and have shown promise for predicting immunotherapy response and differentiating pseudoprogression (Aerts et al., 2014; Trebeschi et al., 2019), though standardization across institutions remains an open problem.

### 6.4 Minimal residual disease in hematologic malignancies

The most mature examples of dynamic biomarker-guided therapy come from hematologic cancers. MRD assessment is now integrated into treatment algorithms for ALL, multiple myeloma, and CML (Berry et al., 2017; Munshi et al., 2017; Hughes et al., 2010). In CML, serial BCR-ABL monitoring guides both escalation and de-escalation, representing a genuine closed-loop dynamic biomarker system. The solid-tumor field can learn from hematologic MRD in both assay design and regulatory adaptation.

### 6.5 Adaptive therapy and ecological intervention

Adaptive therapy broadens the translational picture beyond molecular biomarkers. The ecological motifs described in Section 4.4 have already informed prospective treatment design in metastatic prostate cancer, where abiraterone dosing was modulated to preserve competition from drug-sensitive populations (Gatenby et al., 2009; Zhang et al., 2017; Zhang et al., 2022). The principle is not that adaptive therapy is already generalizable across cancers, but that quantitative ecosystem models can be used to redesign therapy schedules around evolutionary burden rather than maximal cell kill.

### 6.6 Regulatory and equity constraints

Regulatory and equity constraints deserve explicit attention. Existing companion-diagnostic frameworks, particularly in the United States, were built primarily around binary or categorical biomarkers tied to fixed treatment decisions rather than rate-of-change thresholds, trajectory classes, or instability metrics. It is important to distinguish clinical validity—the ability of a biomarker to predict an outcome—from clinical utility, which additionally requires that acting on the biomarker improves patient management; many dynamic biomarkers have achieved validity without yet demonstrating utility. The FDA’s 2014 *In Vitro* Companion Diagnostic Devices guidance and subsequent approvals infrastructure remain essential, but they do not yet map naturally onto continuously updated dynamic signals (Lee and Shen, 2015; Kadam et al., 2026). In Europe, the IVDR has been applicable since 26 May 2022 with phased rollout to support transition, but dynamic multicomponent biomarkers still fit awkwardly within a regulatory logic built for more static analytic claims (Molenkamp et al., 2026; Kahles et al., 2023).

The regulatory pathway most relevant to dynamic and AI-derived biomarkers is, however, beginning to take shape, and it differs in kind from the static companion-diagnostic model. The central obstacle for a continuously updated signal is that conventional authorization certifies a fixed analytic claim, whereas an adaptive algorithm is designed to change after deployment. The U.S. response has been the predetermined change control plan (PCCP): under Section 515C of the Federal Food, Drug, and Cosmetic Act, added by the Food and Drug Omnibus Reform Act of 2022, a manufacturer may specify in advance the modifications an algorithm is permitted to undergo, together with the protocols for validating and implementing them, so that pre-authorized updates do not each require a new marketing submission. The FDA finalized guidance operationalizing this mechanism for artificial-intelligence-enabled device software functions in December 2024, and in August 2025 issued joint guiding principles with Health Canada and the United Kingdom’s MHRA emphasizing that such plans be focused, risk-based, and monitored across the total product life cycle (U.S. Food and Drug Administration, 2024). This framework is the closest existing analogue to regulating a moving target, and it directly addresses the change-control problem that static companion-diagnostic approvals cannot. Two limitations bear on the present review, however. First, PCCPs were designed for software-implemented device functions whose outputs are validated against a defined reference, not for trajectory-class, rate-of-change, or instability biomarkers whose very endpoints—as noted above—are not yet standardized. Second, pre-authorizing the space of permitted modifications presumes that the relevant performance can be monitored in real time, which reintroduces the observability and identifiability constraints emphasized throughout this review. The regulatory machinery for adaptive biomarkers is therefore no longer absent, but it is not yet matched to the specific class of dynamic signals that a systems view of cancer would prioritize.

Equity is not a separate afterthought to this picture but a direct consequence of how dynamic biomarkers are measured and modeled, and it enters through two distinct channels. The first is access to serial measurement itself. A dynamic biomarker is only informative if it can be sampled repeatedly, which presupposes infrastructure—recurring phlebotomy and assay availability, reliable follow-up, and reimbursement for monitoring rather than for a single diagnostic test—that is distributed unevenly across health systems and populations. A standard that rewards dense longitudinal sampling can therefore widen outcome disparities even when the underlying assay is nominally available, which is why equitable-implementation frameworks for liquid biopsy emphasize access and standardization as first-order design constraints rather than downstream considerations (Febbo et al., 2024; Brenner and Fang, 2024). The second channel is representational. Learning-based dynamic models inherit the composition of their training data and the proxies chosen for their labels; a model trained predominantly on one population, or optimized against a convenient but biased proxy for clinical need, can encode and amplify existing disparities while appearing accurate on aggregate metrics, as has been demonstrated for a widely deployed clinical risk algorithm (Obermeyer et al., 2019). Both channels connect equity back to the central themes of this review: representativeness is an observability problem, and an unrepresentative training distribution is a hidden form of partial observation that no amount of model sophistication can correct after the fact.

A further translational challenge is trial design. Dynamic biomarkers demand architectures that accommodate serial measurement, adaptive randomization, and biomarker-triggered decision points—features that exceed the design of conventional parallel-group trials. Adaptive platform designs such as I-SPY 2 and STAMPEDE have demonstrated feasibility (Park et al., 2016; Gilson et al., 2017), but these platforms are operationally complex and introduce statistical challenges that are not yet standardized. For trajectory-based biomarkers, endpoints may need to evolve beyond conventional time-to-event measures.

Taken together, dynamic oncology is no longer only a theoretical aspiration—it has entered the era of selective interventional evidence. But success depends on alignment between the biomarker (detecting a meaningful transition), the intervention (targeting the driving layer), and the health-system context (supporting serial measurement and equitable action).

## 7 Open questions and future directions

Several unresolved questions now define the research frontier. First, what counts as a meaningful dynamic state? Not every fluctuation is a transition, and not every transition is clinically relevant. Future work needs stronger links between mathematical definitions of metastability or instability and biological definitions of persistence, commitment, and reversibility (Wiley et al., 2025; Tirosh and Suva, 2024).

Second, how much data is required for defensible dynamic inference? Many models will remain underdetermined unless their ambition is constrained. Before fitting ODE or SDE models to sparse clinical series, investigators should verify structural identifiability (Villaverde et al., 2016), assess practical identifiability (Raue et al., 2009), and determine whether the sampling schedule is informative. Naive fitting of high-dimensional stochastic models to sparse quarterly measurements is unlikely to yield clinically transportable estimates.

This data-and-identifiability question is exactly what the emerging field of cancer patient digital twins seeks to operationalize. A digital twin in this setting is a patient-specific, continuously updated computational model intended to simulate disease trajectory and treatment response, and it is the most concrete current attempt to instantiate the kind of latent-state, partially observed dynamic modeling this review describes. The effort is institutionally serious: beginning in 2020, the U.S. National Cancer Institute and Department of Energy launched a coordinated set of cancer-patient-digital-twin pilot projects spanning multiscale modeling, treatment-response prediction, and adaptive monitoring of resistance (Stahlberg et al., 2022). Regulatory bodies have grown correspondingly receptive: although no agency yet operates a dedicated digital-twin program, the FDA recognizes model-credibility frameworks such as ASME V&V 40 and has fostered model-informed drug development, while both the FDA and EMA have signaled openness to *in silico* evidence, particularly where conventional trials are infeasible. The honest status of the field, however, matches the caution urged above: a recent scoping review found that most oncology digital- and virtual-twin solutions to date rely on synthetic or limited real-world data, integrate real-time data and machine learning only rarely, and achieve only partial credibility under formal assessment (Ştefănigă et al., 2024). Digital twins therefore represent the clearest translational expression of dynamic-systems oncology and, simultaneously, a concrete demonstration of why identifiability, data sufficiency, and validation—not modeling ambition alone—remain the binding constraints.

Third, can critical-slowing-down-style early-warning signals become useful in oncology? Methods developed in ecological settings (Scheffer et al., 2009; Dakos et al., 2012) and applied to cell-fate transitions (Mojtahedi et al., 2016) suggest that variance and autocorrelation become informative near state destabilization, but clinical cancer data present additional challenges: signals may be confounded by therapy-driven nonstationarity, and careful null models are essential.

Fourth, how should learning-based methods be evaluated dynamically? The relevant questions are whether a method improves latent-state estimation, transition detection, and intervention choice under partial observability (Yates and Van Allen, 2025; Riaz et al., 2025). Foundation models will need frameworks that assess temporal coherence and perturbation robustness, not only annotation accuracy.

Fifth, can dynamic biomarkers be prospectively validated at scale while remaining equitable? Level 3 evidence will require intervention trials, regulatory adaptation, and health-system investment—not only better algorithms or assays (Tie et al., 2022; Tie et al., 2025a; Febbo et al., 2024). The hematologic MRD experience (Section 6.4) suggests that closed-loop dynamic biomarker systems are feasible but require sustained commitment to standardization and access.

## 8 Conclusion

The main argument of this review is straightforward. Precision oncology has inherited a largely static biomarker logic, but the disease it seeks to manage is dynamic, stochastic, and multiscale. Cancer progression, metastasis, treatment adaptation, dormancy, and relapse emerge from coupled behavior across molecular, cellular, spatial, ecological, metabolic, and mechanobiological layers, and they are observed only indirectly through longitudinal clinical measurements.

The contribution of this article is primarily synthetic: it joins stochastic state-space models, ecological motifs, and dynamic biomarkers into a coherent framework for thinking about cancer as a partially observed dynamic disease in which noise is not incidental but constitutive.

If the next decade of oncology is to move beyond static classification, it will need better observability, mechanistically anchored models, responsible use of machine learning, and prospective tests of dynamic biomarkers linked to the right intervention. A systems biology of cancer dynamics will succeed by using mathematics and computation to make biological change more measurable, interpretable, and actionable.
